# Light-sheet fluorescence imaging charts the gastrula origin of vascular endothelial cells in early zebrafish embryos

**DOI:** 10.1038/s41421-020-00204-7

**Published:** 2020-10-27

**Authors:** Meijun Pang, Linlu Bai, Weijian Zong, Xu Wang, Ye Bu, Connie Xiong, Jiyuan Zheng, Jieyi Li, Weizheng Gao, Zhiheng Feng, Liangyi Chen, Jue Zhang, Heping Cheng, Xiaojun Zhu, Jing-Wei Xiong

**Affiliations:** 1 Beijing Key Laboratory of Cardiometabolic Molecular Medicine, Institute of Molecular Medicine and State Key Laboratory of Natural and Biomimetic Drugs, Peking University, Beijing 100871, China; 2 Academy for Advanced Interdisciplinary Studies, Peking University, Beijing 100871, China; 3 School of Engineering, Peking University, Beijing 100871, China

**Keywords:** Developmental biology, Cell biology

## Abstract

It remains challenging to construct a complete cell lineage map of the origin of vascular endothelial cells in any vertebrate embryo. Here, we report the application of in toto light-sheet fluorescence imaging of embryos to trace the origin of vascular endothelial cells (ECs) at single-cell resolution in zebrafish. We first adapted a previously reported method to embryo mounting and light-sheet imaging, created an alignment, fusion, and extraction all-in-one software (AFEIO) for processing big data, and performed quantitative analysis of cell lineage relationships using commercially available Imaris software. Our data revealed that vascular ECs originated from broad regions of the gastrula along the dorsal–ventral and anterior–posterior axes, of which the dorsal–anterior cells contributed to cerebral ECs, the dorsal–lateral cells to anterior trunk ECs, and the ventral–lateral cells to posterior trunk and tail ECs. Therefore, this work, to our knowledge, charts the first comprehensive map of the gastrula origin of vascular ECs in zebrafish, and has potential applications for studying the origin of any embryonic organs in zebrafish and other model organisms.

## Introduction

Determining cell lineage relationships is one of the most fundamental questions in the fields of developmental biology and genetics. An elegant study has charted the lineage tree of *Caenorhabditis elegans* with all the cleavages from the one-cell stage to the adult worm^1^. Cell lineage tracing methods fall into two major categories: optical tracing by dye injection, transgenic reporters, or tissue-specific genetic recombination reporters, and sequence-based lineage tracing by either viral barcodes or genome-editing barcodes with CRISPR/gRNA^2,3^. Due to the regulatory complexity of the lineages and intermingling cell division and migration, it remains challenging to apply these methods to the construction of complete cell lineage trees of the organs of early embryos at single-cell resolution in any vertebrate species.

In zebrafish embryos, as in other vertebrates, hematopoietic and endothelial cells (ECs) arise in close association and are thought to be derived from the ventral mesoderm^4,5^. Single-cell resolution fate maps of the late blastula and gastrula with 2,3-dimethyl-2,3-dinitrobutane-caged fluorescein dextran provided the very first in vivo evidence that individual cells give rise to both hematopoietic cells and ECs, and that the majority of ECs are derived from the ventral–lateral (VL) region of the shield embryo in zebrafish^6^. However, a comprehensive lineage map of the origin of vascular ECs in vertebrates has not yet been reported.

Advanced optical live-imaging methods have shed new light on approaching this goal; among these, light-sheet fluorescence microscopy (LSFM) has advantages such as low photo-toxicity, rapid imaging, and a capacity for long-term three-dimensional imaging^7^. In toto imaging of the early stages of fly, zebrafish, and mouse embryos at single-cell resolution has now been reported^7–9^. Recently, LSFM has been successfully applied to documenting neuronal cell lineages, movements, and activities in the entire spinal cord of live zebrafish embryos^10^. Although it often has superior performance, LSFM is not well used as traditional imaging methods because of the ‘do-it-yourself ethic’ and the problem of big data^11^. In this study, we used LSFM to acquire and resolve in toto images of early zebrafish embryos, and charted a comprehensive origin map of all vascular ECs at single-cell resolution — this was made possible by a combination of Zeiss Z.1 LSFM (Carl Zeiss, Jena, Germany), commercially available Imaris software, and the user-friendly image *a*lignment, *f*usion, and *e*xtraction all-*i*n-*o*ne (AFEIO) software that we created. Furthermore, this LSFM system together with the AFEIO software makes it possible to construct the cell-lineage trees of other organs in zebrafish and other model organisms.

## Results

### Optimized mounting and dual-view light-sheet microscopy imaging

**Fig. 1:**
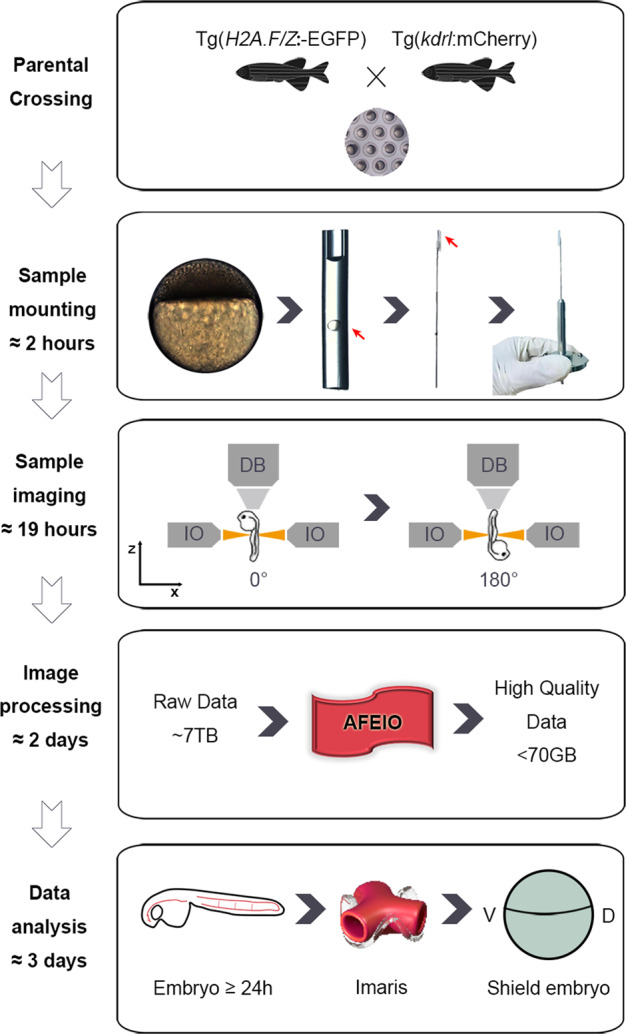
Flowchart of high-resolution imaging of zebrafish embryos with a Zeiss light-sheet fluorescence microscope. *Parental crossing*: collection of transgenic embryos from crosses between Tg(*H2A.F/Z*:EGFP) and Tg(*kdrl*:mCherry) transgenic lines. *Sample mounting***:** careful de-chorionation of embryos at 4 hpf and their transfer into a fluorinated ethylene propylene tube filled with 0.2% agarose. The tube is fixed to a fine wire and then mounted to a holder. *Sample imaging***:** The illumination objectives (IOs) illuminate the sample from the left and right alternately, while the detection objective (DB) detects signals at 0°. The embryo is then rotated and the 180° images are acquired in the same way. *Image processing***:** The raw data (~7 TB) are processed using the AFEIO software to obtain fused high-resolution data (~70 GB). *Data analysis***:** The processed data are imported into Imaris software to run retrospective lineage analysis and determine a gastrula map of the origin of vascular ECs from 6 to 27 hpf.

To establish the gastrula origin of vascular ECs, we took advantage of LSFM to retrieve full coverage of all developmental cell divisions and migrations in Tg(*H2A.F/Z*:EGFP) transgenic embryos^7,9^ along with the Tg(*kdrl*:mCherry) transgenic reporter for locating vascular endothelial progenitors/cells. Tg(*H2A.F/Z*:EGFP)^tg/+^; Tg(*kdrl*:mCherry)^tg/+^ double transgenic embryos were used for all imaging experiments (Fig. 1). It has been reported that specimen rotation combined with multiview imaging decreases the degradation of imaging quality induced by tissue scattering and absorption^12^, as well as improves the axial optical resolution by compositing with an isotropic point-spread-function^13^. On the other hand, specimen rotation requires high mechanical stability, which can be achieved by using a 1% or higher agarose matrix. A previous study reported that 0.4% or higher agarose interferes with embryo growth and mobility while fluorinated ethylene propylene (FEP) with low concentrations of agarose is both optically clear and sufficiently confines the living embryo in a physiological environment, thus providing the optimal choice for live imaging^14^. Early zebrafish embryos frequently twitch in the absence of tricaine when they are mounted with their intact chorion inside an E3-filled FEP tube^15^. However, prolonged exposure to tricaine suppresses the contraction of cardiac, skeletal, and smooth muscles and thus affects the hemodynamics^16,17^. To decrease the effect of both chorion and tricaine, we optimized the mounting method by embedding dechorionated embryos in 0.2% low-melting-point agarose in FEP to guarantee both mechanical stability and normal embryonic development, as well as to ensure the exchange of oxygen and fluids during imaging. Tricaine and other drugs, if needed, could be added to the E3 culture medium at any time (Fig. 1).

**Fig. 2:**
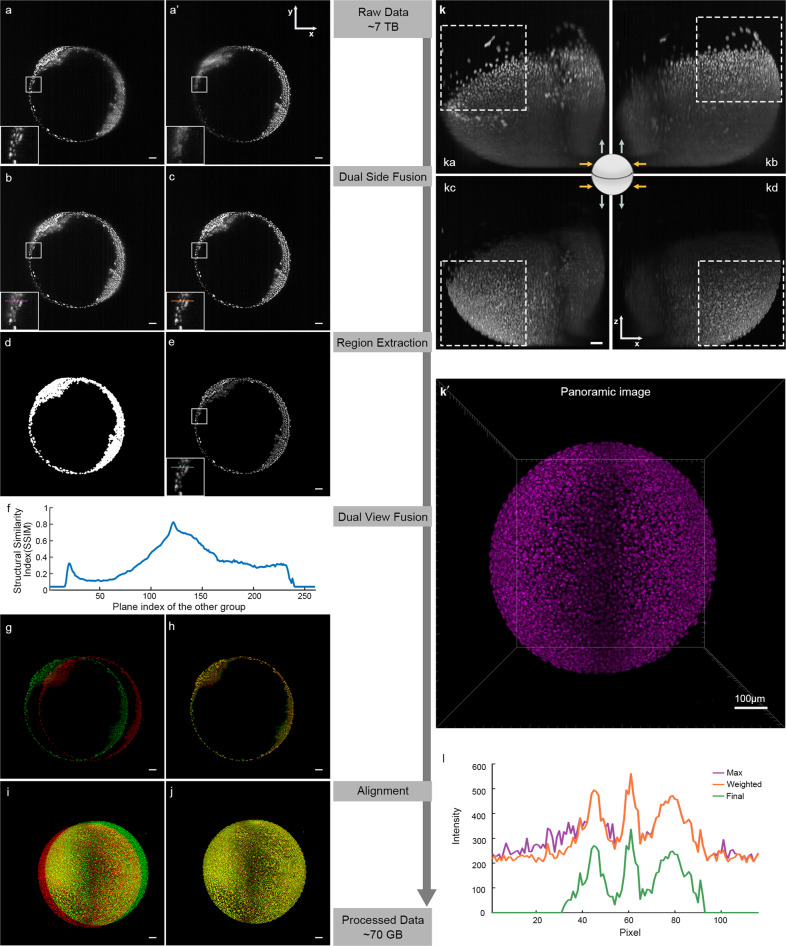
Single-cell resolution images of zebrafish embryos constructed from light-sheet data using AFEIO. The compressed, high-quality imaging data (~70 GB) are derived from the original raw data (~7 TB) using the AFEIO workflow, consisting of dual-side fusion, region extraction, dual-view fusion, and alignment. **a**, **a′** Original images of the same plane illuminated from the left (**a**) and right (**a**′). **b** Original image from the bi-directional illumination processed with ‘Max’ fusion. Enlarged square area refers to **l** for the intensity distribution along the purple line. **c** Original image from the bi-directional illumination processed with ‘Weighted’ fusion. Enlarged square area refers to **l** for the intensity distribution along the orange line. **d** The extraction mask, which only contains the pixels with information. **e** The image extracted from the ‘Weighted’ fusion image after masking. Enlarged square area refers to **l** for the intensity distribution along the green line. **f** Structural similarity of the selected plane from the 0° group and all planes from the 180° group. **g**, **h** A selected plane from the 0° group (red) and the most similar plane in the 180° group (green) (**g**) along with overlap after registration (**h**). **i**, **j** The shift between different time points (**i**) and overlap after registration (**j**). **k** Raw image projections of stacks from the top view with different illumination and imaging directions (orange arrows, illumination directions; green arrows, imaging directions). Illumination from the left and detection at 0° (**ka**); illumination from the right and detection at 0° (**kb**); illumination from the left and detection at 180° (**kc**); illumination from the right and detection at 180° (**kd**). **k**′ Output of the fused imaging data (also shown in Supplementary Movie S4). **l** Intensity distribution of maximal fusion, sigmoidal weighted fusion, and the final image; note that compared to the maximal fusion, the sigmoidal weighted fusion markedly improved the image contrast (Supplementary Fig. S3c). Scale bars, 50 μm (**a**–**e**, **g**–**k**) and 100 μm (**k**′).

To construct single-cell resolution images of a zebrafish embryo, it is essential to capture light-sheet images from different viewing directions. The mounted embryo was illuminated first on the left side and then on the right side, images were acquired via z-stacks from 0°, and the embryo was then rotated and the images were acquired from 180° (Fig. 1), thus generating four sets of imaging data at each time point (Fig. 2a, a′ and k). In this work, we captured three sets of image data from three embryos: embryo #1 (6–27 hpf), embryo #2 (6–22 hpf), and embryo #3 (7–27 hpf) (Supplementary Movies S1–S3). The raw data were ~5–7 terabytes (TB) in size (Supplementary Fig. S1). To fuse the data acquired by Zeiss Light Sheet Z.1, we first used the self-contained fusion module of Zen, the supporting software of Z.1. The Zen module is based on the image view settings on the microscope, but was not able to handle the two-view imaging (0° and 180°) of our samples and the imaging speed was too slow to keep up with the rapid development of zebrafish embryos. Moreover, the Zen module performed poorly on four-view fusion (Supplementary Fig. S2). In addition, we found that for dual-illumination side-fusion, the only supportive strategies available were maximal, mean, and Fourier domain maximal, but all these measurements decreased the contrast of images. To address these questions, we created a software named AFEIO as will be described in “Materials and methods” section (Fig. 2). We performed dual-side fusion (Fig. 2b, c), regional extraction (Fig. 2d, e), dual-view fusion (Fig. 2f–h), and alignment (Fig. 2i, j) in the Tg(*H2A.F/Z*:EGFP) channel. In addition, in the Tg(*kdrl*:mCherry) channel, we used a threshold based on a region where the signal intensity in the nuclei was strong (Supplementary Fig. S3a, a′ and b). Together, we were able to obtain time-lapse, shift-corrected, fused-in-whole panoramic image stacks of the embryos (Fig. 2k′; Supplementary Movies S4 and S5).

### AFEIO software for time-lapse and dual-view image processing

It has been reported that the sigmoidal weighting function can be applied to dual-side fusion^18^. Compared with maximal fusion, we found that sigmoidal weighted fusion significantly improved the image contrast (Fig. 2l; Supplementary Fig. S3c). After background subtraction, an extraction mask was applied to the image, and only the regions where there were signals were retained (Fig. 2d, e). With advances in run-length encoding, the volume of data was significantly reduced in this step. In addition, Huisken and colleagues have already shown the feasibility of fusing light-sheet images taken from different directions^19^. Given that the interval between capturing images from two view directions was very short compared with the duration of rapid embryonic development in zebrafish, we regarded them as occurring simultaneously. Furthermore, there was sufficient overlap in the middle part, sharing the same spatial information, and this was used for static registration. Thus, we used the structural similarity index (Fig. 2f) to match the fused images corresponding to the same plane from the two directions^20^. Finally, to correct displacement at different time points, we first projected the main view and left view of a single embryo, then used the Fourier–Merlin transform to calculate the total displacement of the embryo in three dimensions between different time points, and corrected the displacement to achieve alignment (Fig. 2i, j). This image processing can be done on a personal computer equipped with a single i7–7700 CPU, 8 GB DDR3 memory, a 256 GB SSD, and a data-saving mobile hard disk. In addition to processing the datasets acquired from the Zeiss Z1 system, the AFEIO software and strategy were also able to process datasets from other light-sheet microscopies such as Luxendo Muvi-SPIM (Supplementary Movie S6). This processing strategy provided a high-contrast, low-redundancy dataset, reducing ~7 TB of raw data to ~70 GB for each of the three embryos (Supplementary Fig. S1) for further single-cell tracking of the origin of ECs during early development.

### Retrospective lineage tracing of the origin of vascular ECs

The ~70 GB of high-quality data was then imported to the software ‘Imaris’ to calculate cell lineage relationships. We tracked the lineages of all EGFP^+^ cells and then filtered out EGFP^+^/mCherry^+^ ECs. The nuclei of the cells were labeled with Tg(*H2A.F/Z*:EGFP) and detected as spots by Imaris. The detailed locations of spots with tracks allowed us to follow each cell of a developing embryo (Supplementary Movie S7). Statistical analysis of embryo #1 showed that the total number of cells doubled from 8176 (6 hpf) to 18,698 (27 hpf) during early development (Supplementary Fig. S4). The number of cells at 18 hpf was 15,905, close to that in a previous report^9^, further validating the feasibility of our method. The total numbers of cells in embryo #2 were similar from 6 to 22 hpf (Supplementary Fig. S4). Therefore, these results suggest that our method is able to resolve in toto imaging of a zebrafish embryo consisting of > 18,000 cells at single-cell resolution.

**Fig. 3:**
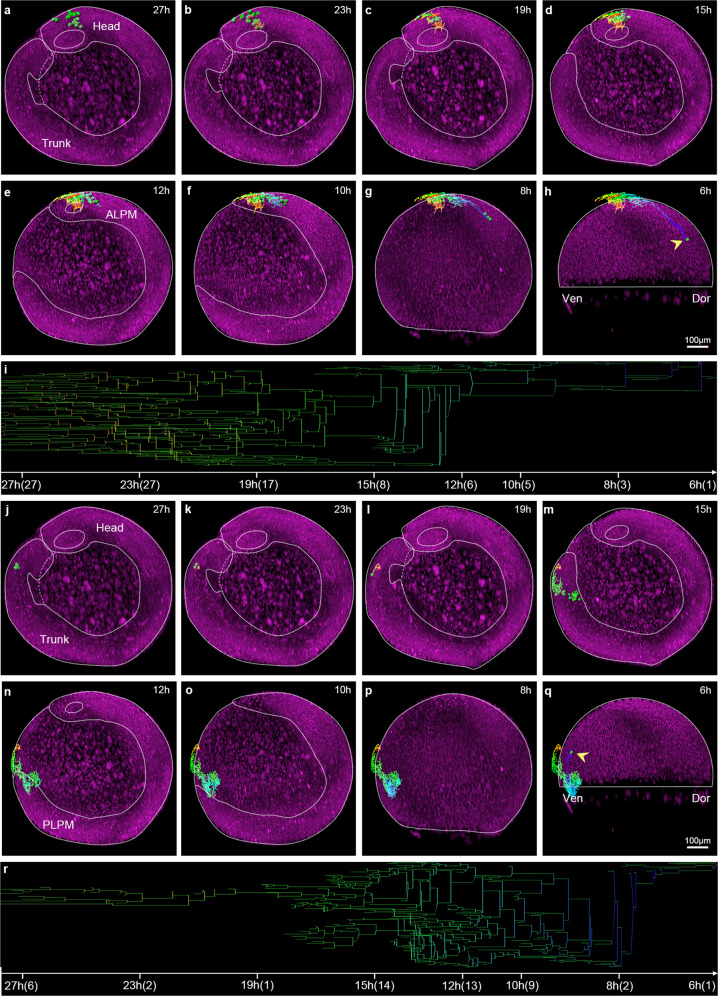
Retrospective cell-lineage tracking reveals the distinct gastrula origins of vascular ECs in the trunk and head. **a**–**h** Representative images showing vascular ECs of the head (green) at 27 hpf (**a**) retrospectively tracked back to a dorsal cell at 6 hpf (**h**, arrowhead), with ECs in the head from 23 to 15 hpf (**b**–**d**), the ALPM at 12 hpf (**e**), and the dorsal cells at 10 and 8 hpf (**f**, **g**). **i** Lineage tree map showing that the selected brain ECs at 27 hpf are derived from a dorsal cell at 6 hpf. Along the time line, 27 h (27), 27 ECs at 27 hpf; 23 h (27), 27 ECs at 23 hpf, etc. **j**–**q** Representative images showing vascular ECs of the trunk at 27 hpf (**j**) retrospectively tracked back to a ventral cell at 6 hpf (**q**), with ECs in the trunk from 23 to 15 hpf (**k**–**m**), the PLPM at 12 hpf (**n**), and the ventral cells at 10 and 8 hpf (**o**, **p**). **r** Lineage tree map showing that the selected trunk ECs at 27 hpf derive from a single ventral cell at 6 hpf. Along the time line, 27 h (6), 6 ECs at 27 hpf; 23 h (2), 2 ECs at 23 hpf, etc. Yellow arrowhead points to the gastrula cells; multiple-colored lines showing the cell migration tracking; Scale bars, 100 μm.

**Fig. 4:**
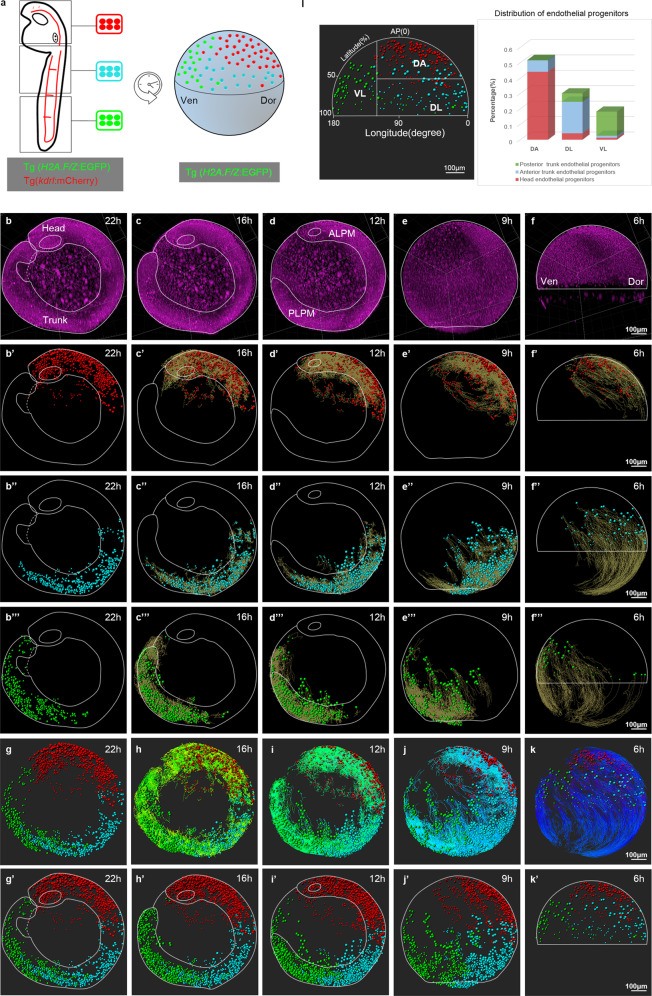
Retrospective cell-lineage tracing creates a comprehensive map of the origin of vascular ECs along the dorsal–ventral (D–V) and anterior–posterior (A–P) axes of the gastrula. **a** Scheme for retrospective lineage tracing of the origin of vascular ECs using Tg(*H2A.F/Z*:EGFP) to label the nuclei of all embryonic cells and Tg(*kdrl*:mCherry);Tg(*H2A.F/Z*:EGFP) to co-label the nuclei of all ECs. All mCherry^+^ nuclei are classified with different colors: red in the head (the otic vesicle as the boundary between head and trunk), blue in the anterior trunk, and green in the posterior trunk. The gastrula progenitors at 6 hpf are retrospectively tracked from ECs at different locations at 22 hpf with Imaris software. Dor dorsal, Ven ventral. **b**–**f** Developmental morphology of embryo #1 at 22 hpf (**b**), 16 hpf (**c**), 12 hpf (**d**), 9 hpf (**e**), and 6 hpf (**f**) (note the bend tail at 22 hpf). **b′**–**f′** Retrospective lineage tracing from the head ECs at 22 hpf (**b′**), 16 hpf (**c’**), 12 hpf (**d’**), and 9 hpf (**e’**) to the DA gastrula cells at 6 hpf (**f’**) (dynamic lineage tracking is shown in Supplementary Movie S10). **b”**–**f”** Retrospective lineage tracking from the anterior trunk ECs at 22 hpf (**b”**), 16 hpf (**c”**), 12 hpf (**d”**), and 9 hpf (**e”**) to the DL gastrula cells at 6 hpf (**f”**) (dynamic lineage tracking in Supplementary Movie S11). **b”’**–**f”’** Retrospective lineage tracking from posterior trunk ECs at 22 hpf (**b”’**), 16 hpf (**c”’**), 12 hpf (**d”’**), and 9 hpf (**e”’**) to VL gastrula cells at 6 hpf (**f”’**) (dynamic lineage tracking in Supplementary Movie S12). **g**–**k** Retrospective lineage tracking showing the distribution of the three clusters of ECs (red, blue, and green) with cell-lineage tracking lines (multiple-colored lines) from 22 hpf (**g**), 16 hpf (**h**), 12 hpf (**i**), and 9 hpf (**j**) to the gastrula progenitors at 6 hpf (**k**) (dynamic tracking in Supplementary Movie S14). **g’**–**k’** Retrospective lineage tracking showing the distribution of the three clusters of ECs (red, blue, and green) without cell-lineage tracking lines from 22 hpf (**g’**), 16 hpf (**h’**), 12 hpf (**i’**), and 9 hpf (**j’**) to the gastrula progenitors at 6 hpf (**k’**). Scale bars, 100 μm. **l** Left panel: the gastrula divided into a DA region along the A–P axis (0–100% latitude), as well as DL and VL regions along the D–V axis (0–180° longitude) (AP(0), animal pole as 0% latitude; scale bar, 100 μm). Right panel: quantitative analysis showing the percentage of the three regions (DA, DL, and VL) that contribute to ECs in the head (red), anterior trunk (blue), and posterior trunk (green). Note that the DA gastrula contributes to the head ECs, DL to the anterior trunk ECs, and VL to the posterior trunk ECs.

To trace the lineages of vascular ECs, we selected one EGFP^+^/mCherry^+^ double-positive nucleus near the eye at 27 hpf while simultaneously marking its sister nucleus (Fig. 3a). Retrospective fate-mapping from 27 to 6 hpf showed that these endothelial descendants in the head (Fig. 3a–d) migrated from the anterior lateral plate mesoderm (ALPM) (Fig. 3e), and originated from a single endothelial progenitor in the dorsal side of the shield embryo (Fig. 3f–h, yellow arrowhead; Supplementary Movie S8). The lineage tree map showed that 27 ECs in the head at 27 hpf were traced back from 27 ECs at 23 hpf, 17 ECs at 19 hpf, 8 ECs at 15 hpf, 6 ECs at 12 hpf, 5 ECs at 10 hpf, 3 ECs at 8 hpf, and a single dorsal endothelial progenitor at 6 hpf, suggesting dynamic cell division and death during early development (Fig. 3i). In parallel, we selected one EGFP^+^/mCherry^+^ double-positive nucleus in the posterior trunk at 27 hpf, and simultaneously marked its sister cells (Fig. 3j). Retrospective fate mapping from 27 to 6 hpf showed that their endothelial descendants (Fig. 3j–m) migrated from the posterior lateral plate mesoderm (PLPM) (Fig. 3n), and traced back to one endothelial progenitor in the ventral side of the shield embryo (Fig. 3o–q, yellow arrowhead; Supplementary Movie S9). The lineage tree map revealed that six selected ECs of the posterior trunk at 27 hpf were traced back from 2 ECs at 23 hpf, 1 ECs at 19 hpf, 14 ECs at 15 hpf, 13 ECs at 12 hpf, 9 ECs at 10 hpf, 2 ECs at 8 hpf, and a single ventral endothelial progenitor at 6 hpf (Fig. 3r). To construct a comprehensive lineage map on the origin of all vascular ECs, we marked all EGFP^+^/mCherry^+^ double-positive nuclei of ECs with different colors along the anterior–posterior axis from 18 to 22 hpf, and then performed lineage tracing back to the gastrula progenitors using Imaris. Taking the otic vesicle as the boundary between head and trunk, we marked vascular ECs of the head in red, and divided ECs of the trunk into two parts, the anterior trunk marked in blue and the posterior trunk marked in green (Fig. 4a).

We then performed lineage analysis of the three groups of endothelial progenitors using retrospective fate mapping from 22 to 6 hpf, showing the developmental morphology at different stages (Fig. 4b–f; Supplementary Fig. S6 and Movie S1). The head vascular ECs from 22 hpf were traced back mainly to the ALPM at 12 hpf (Fig. 4b’–d’; Supplementary Fig. S6a’–c’) and the dorsal–anterior (DA) gastrula progenitors near the animal pole at 6 hpf (Fig. 4d’–f’; Supplementary Movie S10 and Fig. S6c’–e’). The anterior trunk vascular ECs at 22 hpf were traced back to both the ALPM and PLPM at 12 hpf (Fig. 4b”–d”; Supplementary Fig. S6a”–c”) and the dorsal–lateral (DL) gastrula progenitors at 6 hpf (Fig. 4d”–f”; Supplementary Movie S11 and Fig. S6c”–e”). The posterior trunk vascular ECs at 22 hpf were traced back to the PLPM at 12 hpf (Fig. 4b”’–d”’; Supplementary Fig. S6a”’–c”’) and the VL gastrula progenitors at 6 hpf (Fig. 4d”’–f”’; Supplementary Movie S12 and Fig. S6c”’–e”’). In addition, the vascular EC migration and division tracks were shorter between 22 and 12 hpf than those between 12 and 6 hpf, suggesting rapid derivation, division, and migration of ECs along the body axes from 6 to 12 hpf (Supplementary Movie S13, yellow lines). The combined lineage maps of all three groups of endothelial progenitors were projected onto representative images with lineage tracing lines (Fig. 4g–k; Supplementary Fig. S6f–j) or without them (Fig. 4g’–k’; Supplementary Fig. S6f’–j’) as well as movies (Supplementary Movies S14, S15). The lineage tree maps of all ECs from embryo #1 were constructed in the scalable vector graphics (SVG) format and were included as a supplementary file. Quantitative analysis of two sets of data from embryos #1 and #2 revealed that vascular ECs derived from broadly distributed endothelial progenitors of the shield embryo (Fig. 4l, left panel from embryo #1; Supplementary Fig. S6j’ from embryo #2). The dorsal–anterior (DA) gastrula contributed to the formation of ECs mainly in the head and seldom in the anterior trunk; the DL gastrula gave rise to ECs mainly in the anterior trunk and seldom in the posterior trunk and head; and the VL gastrula contributed to ECs mainly in the posterior trunk (Fig. 4l; right panel from embryo #1). Therefore, in contrast to the previous report that vascular ECs are primarily derived from the VL gastrula cells^5,6^, our results support the conclusion that endothelial progenitors are distributed throughout the gastrula along the dorsal–ventral and anterior–posterior axes (Fig. 4k, k’; Supplementary Fig. S6j, j’); the DA gastrula cells give rise to most of the ECs in the head, while the DL and VL gastrula cells contribute to most of the ECs in the trunk and tail.

Importantly, this digital cell-lineage mapping enabled the visualization of all cell divisions, migration, and differentiation at single-cell resolution. We randomly selected a single cell near the animal pole of the shield embryo (Supplementary Fig. S7a), which started to rapidly divide and migrate upwards to the animal pole and then migrated to the right (Supplementary Fig. S7b). At ~9 hpf, its descendants began to migrate back (Supplementary Fig. S7c, d) and further migrated to the upper left of the starting position (Supplementary Fig. S7e, f), finally reaching the dorsal head between the eyes (Supplementary Fig. S7g, h, Movie S16). The lineage tree map showed dynamic cell division, migration and death from 6 to 27 hpf (Supplementary Fig. S7i). Thus, if the cells of interest in embryonic organs are marked with a fluorescent reporter, the method described here enables tracing them back to their gastrula origin in early zebrafish embryos.

### Prospective lineage tracing of the origin of vascular ECs by using Kaede reporter

To confirm the distinct gastrula origins of vascular ECs between the head and trunk/tail, we used the photo-convertible fluorescent protein Kaede to analyze prospective cell lineages as previously reported^21^. We first established a Tg(*kdrl*:kaede) transgenic zebrafish line in which *kaede* was driven by the *kdrl* promoter. We generated several independent Tg(*kdrl*:kaede) transgenic lines from different F0 founders and all of them had similar kaede expression patterns, with quite strong expression at 6 hpf and later in blood vessels. Whole-mount in situ hybridization and semi-quantitative PCR revealed that kdrl was expressed during gastrulation (4 hpf at the earliest) and several vascular endothelial growth factor (VEGF) ligands were also expressed in early embryos (Supplementary Fig. S8), which is consistent with previous reports in zebrafish^22^ and in mice^23,24^. In addition, kaede expression in the ALPM in Tg(*kdrl*:kaede) transgenic embryos partly overlapped with that in Tg(*scl-α*:dsRed) embryos (Supplementary Fig. S9)^25^. These data suggest that Tg(*kdrl*:kaede) recapitulates the endogenous *kdrl* expression pattern. Upon photoactivation by a 405-nm laser, a small cluster of Kaede^+^ cells in the dorsal gastrula at 6 hpf were converted from fluorescent green to red (Supplementary Fig. S10a). The development of these red cells and the other green Kaede^+^ cells were followed by confocal microscopy at 12, 20, and 28 hpf (Supplementary Fig. S10b–d). We noted that the red cells divided and migrated to the ALPM at 12 hpf (Supplementary Fig. S10b), continued to migrate to the head region, and eventually formed vascular endothelium there (Supplementary Fig. S10c, d). In contrast, when a small group of ventral gastrula cells were photoactivated from Kaede^+^ fluorescent green to red (Supplementary Fig. S10e), these red cells divided and migrated to the PLPM at 12 hpf (Supplementary Fig. S10f), then continued to migrate, and eventually formed vascular ECs in the trunk/tail (Supplementary Fig. S10g, h). Therefore, these data further support the conclusion that vascular ECs are derived from different gastrula cells along the body axis, with the fate of VL gastrula cells being ECs in the trunk/tail and that of DA gastrula cells being ECs in the head.

## Discussion

We have developed an effective workflow for tracing cell lineages in zebrafish using a Zeiss Z.1 LSFM, Imaris software, and AFEIO software, thus making the light-sheet system and big data processing available to biology laboratories. In the field of multiview registration fusion, there are many well-developed algorithms and protocols, by which excellent work has already been reported^26–29^. IsoView image deconvolution software provides a high processing speed, isotropic resolution, and a high compression rate^28^. Mainly based on multiview joint deconvolution, this software performs best on imaging datasets from more than two views, particularly on four views. Others have applied a bead-based registration algorithm, in which the beads act as feature points for image registration^26,30,31^. In fact, we first tried to use feature points for registration, but early zebrafish embryos often lacked consistent and clear features for identifying the feature points. Because the cross-correlation uses all pixels for registration, we applied it to present the global features of the sample together with the structural similarity index for registration, and finally obtained a satisfactory result. In addition, those deconvolution steps often require a huge amount of computation; to mitigate the computational loads, we chose similarity and phase correlation to do multiview fusion only for dual-view fusion, of which the format of our data was acquired. Based on the algorithm we used, our registration method can be upgraded to the fusion of four or more views, only requiring an additional rotation step. Thus, it is conceivable that those who would like to achieve four-view bead-free registration will also benefit from our algorithm, and the current AFEIO enables general users to perform dual-view registration and fusion. Therefore, our AFEIO software builds upon existing imaging registration and fusion software and incorporated additional functionalities to better suit applications for early zebrafish embryos.

**Fig. 5:**
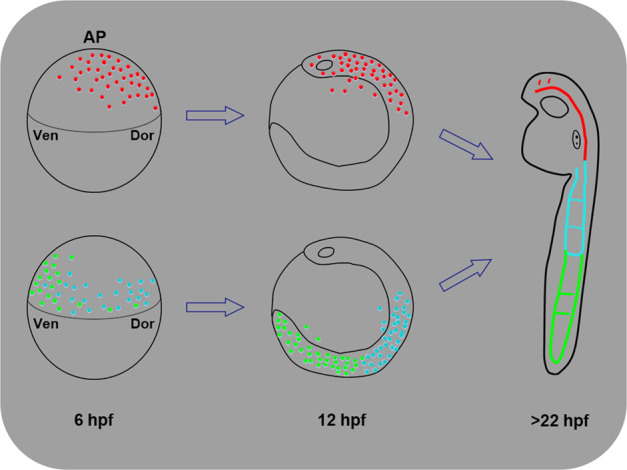
A new lineage-map model of the gastrula origin of vascular ECs in zebrafish. Based on this work, we propose that the DA gastrula cells (red) contribute to brain ECs, the DL gastrula cells (blue) to anterior trunk ECs, and the VL gastrula cells (green) to posterior trunk ECs. This new model suggests that vascular ECs are derived from broad areas of the gastrula and are not limited to the VL gastrula cells. AP animal pole, Dor dorsal, Ven ventral.

To our knowledge, our data reveal the first comprehensive digital map of the origin, division, and migration of ECs in zebrafish embryos from 6 to 27 hpf, elucidating the contribution of the DA gastrula to ECs in the head and of the VL and DL gastrula to ECs in the trunk and tail as summarized in Fig. 5. This new model suggests that vascular ECs are derived from broad areas of gastrula cells and are not limited to the VL gastrula cells^6^. In addition, vascular endothelial progenitors are distributed along the anterior–posterior axis at 12 hpf, suggesting that they were likely not restricted to the ALPM and PLPM, which needs to be investigated in the future. This work is consistent with the notion that the development of cerebral and trunk vessels is regulated by distinct signaling pathways as previously reported^32^. Regarding the major cell-lineage tracing methods, dye injection can only trace a few cell divisions^33^, tissue-specific transgenic reporters or genetic recombination methods are heavily dependent on the availability of the promoters/enhancers required to label both progenitors and descendants^2,34^, and retroviral-based or genome editing-based barcodes are able to trace long-term cell lineage relationships but do not capture spatial information^2,35,36^. Our method resolves both long-term and spatial light-sheet images by only using a transgenic reporter that labels the descendants/differentiated cells of interest, such as the *kdrl*:mCherry-labeled ECs in this work. Together, this work not only deciphers the distinct gastrula origins of cerebral and trunk ECs in zebrafish^37^, but also reveals the potential of our method for broad applications in decoding the origin of organs in zebrafish and other model organisms.

## Materials and methods

### Zebrafish lines and maintenance

Zebrafish were raised and handled in accordance with the animal protocol *IMM-XiongJW-3* approved by the Peking University Institutional Animal Care and Use Committee accredited by the Association for Assessment and Accreditation of Laboratory Animal Care International. The Tg(*kdrl*:kaede) transgenic line was created to express Kaede under the control of the vascular endothelium-specific *kdrl* promoter^38^ using the Tol2 transposon elements^39^ and Tg(*kdrl*:mCherry)^40^ line was obtained from Dr. Bo Zhang (School of Life Science, Peking University, China). The Tg(*H2A.F/Z*:EGFP) line was kindly provided by Dr. Qiang Wang (Institute of Zoology, Chinese Academy of Sciences, China)^41^.

### Preparation of FEP tubes and wire plungers

FEP tubes (S1815-04; Bola, Grünsfeld, Germany) were rinsed sequentially with 1 M NaOH and 0.5 M NaOH, ultrasonicated, rinsed in double-distilled water and 70% ethanol, and ultrasonicated again^14^. Finally, the tubes were cut and stored in 50-mL tubes. After three rinses in double-distilled water, each tube was coated with 3.0% methylcellulose before use. The wire plungers (701998; BRAND) were cleaned with 70% ethanol and stored in an autoclaved container.

### Optimized mounting of zebrafish embryos

Tg(*H2A.F/Z*:EGFP)^Tg/+^; Tg(*kdrl*:mCherry)^Tg/+^ double transgenic zebrafish embryos were collected and maintained at 28.5 °C. At 4 hpf, the embryos were carefully dechorionated with pronase (11458643001; Roche), followed by five washes with E3 medium. Then, each embryo was carefully transferred into 0.2% ultrapure low melting-point agarose (16520-050; Invitrogen) without tricaine. The embryo was then drawn into an FEP tube using a 1-mL syringe with an 18 G blunt needle. We used a razor-blade to cut the tube from the needle, and retained the segment of tube containing the embryo (~1 cm long). After solidification of the agarose, the tube was fixed to a fine wire plunger with glue and Parafilm (PM-996; Bemis, Oshkosh, WI, USA). Before imaging, the extra FEP was cut from both ends to ensure that the tube was filled with agarose and to avoid the formation of bubbles. The tube was cut as short as possible to reduce fluctuations during rotation and guarantee E3 infiltration. The ends of the wire plunger without FEP were wrapped with Parafilm (PM-996; Bemis) and fixed in a capillary (701908; BRAND). Finally, the capillary was fixed in a sample holder for subsequent imaging (Fig. 1).

### Long-term live imaging by LSFM

The images were acquired with a Zeiss Z.1 LSFM, which recorded one-view images in 3-μm Z steps and 260 images per time point, thus taking 30–35 s, which meant that the temporal resolution permitted no more than two viewing directions. We used two 5× illumination objectives, a 10× imaging objective, and 488 and 561-nm lasers. First, we adjusted the longest diameter of the wire plunger in the FEP tube to be perpendicular to the objectives, thus avoiding contact with them during sample rotation and imaging. Second, we adjusted the sheet position and image parameters, and set the image range. The image size was 1920 × 1920 pixels. The step size in the *Z*-axis was 3 μm and 260 images were obtained in one view; the initial view was set as group 1 (0°), then the sample was rotated 180° and set as group 2 (180°). The temperature during imaging was maintained at 28.5 °C. From 6 to 10 hpf, the time interval between views was 90 s and the camera only detected the EGFP signals of the double Tg(*H2A.F/Z*:EGFP); Tg(*kdrl*:mCherry) transgenic embryos. At ~10 hpf, the mCherry signal appeared, thus we turned on the 561-nm laser and the camera simultaneously acquired both EGFP and mCherry signals at 150-s intervals. After 12 hpf, tricaine (200 mg/L) was applied to anesthetize the embryos. The images of embryos from 6 to 27 hpf were acquired and initially stored in a computer workstation for the light-sheet microscope.

### Lineage tracing and statistical analysis with Imaris software

The above processed data were imported into Imaris. First, we performed lineage tracking of all embryonic cells with the EGFP channel. Second, we filtered the mCherry channel co-localization with the EGFP channel to mark ECs and their progenitors. Then we selected a single or several endothelial progenitors of interest to present the track and fate of these cells, and we labeled ECs at different positions with different colors from 15 to 27 hpf. Using the otic vesicle as the boundary between head and trunk, we marked the vascular ECs of the head in red and divided the ECs of the trunk into two parts, anterior marked in blue and posterior marked in green. When this labeling was completed, ECs at the different locations were clearly distinguishable. In the data tab, the numbers of both total embryonic cells and ECs at different time points were exported in Excel format.

### Qualitative analysis of ECs with MATLAB

To qualitatively analyze the numbers of endothelial progenitors at different locations in the dorsal and ventral regions of the gastrula at 6 hpf, we used a MATLAB script. First, we set the size of a single cell as 1 pixel in Imaris and exported the images. Second, we separated the different colors in the original images. The cells were separated into three colors: red, green, and blue, whose RGB values were [255,0,0], [0,255,0], and [0,255,255], respectively. We used *R* = 255 to separate the red cells, *B* = 255 to separate the blue cells, and counted the remaining cells as green. Finally, we counted the numbers of single-color cells. By binarizing the images, we then considered the pixel gray level as 0 where there were no cells and as 1 where cells were present. By adding up the gray levels of the images, we obtained the numbers of cells of each color in the DA, DL and VL regions.

### UV-induced photoconversion of Kaede for fate mapping of vascular ECs

Kaede is fluorescent green but can be photo-converted to fluorescent red by violet or UV light^21^. Tg(*kdrl*:kaede) transgenic embryos with strong fluorescent signals were mounted in 0.35% low-melting-point agarose (Sigma). These embryos were photoactivated using a Nikon A1R microscope (Nikon, Tokyo, Japan), and captured images were processed with the 3D projection feature of NIS-Elements software. Briefly, at 6 hpf, a group of Kaede^+^ cells were activated with a 405-nm laser until their fluorescent green signals were nearly absent. These embryos were then imaged via both the 488 and 561-nm channels at 12, 20, and 28 hpf while anesthetized by tricaine.

## Supplementary information

Supplementary Information

Cell lineage tree maps for all endothelial cells

## Supplementary information

**Supplementary Information** accompanies the paper at (10.1038/s41421-020-00204-7).
