# Supplementary material for: Light-sheet fluorescence imaging charts the gastrula origin of vascular endothelial cells in early zebrafish embryos
Source: Cell Discov. 2020 Oct 27;6:74. doi: 10.1038/s41421-020-00204-7 (PMC7588447; doi:10.1038/s41421-020-00204-7)
Supplement: Supplementary file 1 — Supplementary Information [file 41421_2020_204_MOESM1_ESM.pdf]

Supplementary Information

Light-Sheet Fluorescence Imaging Charts the Gastrula Origin of Vascular Endothelial Cells in Zebrafish Embryos

Meijun Pang[1,†], Linlu Bai[1,2,†], Weijian Zong[1], Xu Wang[1], Ye Bu[1], Connie Xiong[1], Jiyuan Zheng[1], Jieyi Li[1], Weizheng Gao[3], Zhiheng Feng[3], Liangyi Chen[1], Jue Zhang[3], Heping Cheng[1], Xiaojun Zhu[1,*], and Jing-Wei Xiong[1,2,*]

Correspondence to: jingwei_xiong@pku.edu.cn or zhuxiaojun@pku.edu.cn

The List of Supplementary Information:

Supplementary Figs. S1 to S10, Supplementary Movies S1 to S16, AFEIO software and its user instruction, and a file of cell-lineage maps for all endothelial cells in the SVG format can be downloaded from:

https://disk.pku.edu.cn:443/link/4ED92786A171450C943A370BBC545007

AFEIO software and its user instruction can also be downloaded on the Github website:

https://github.com/Manearth/AFEIO

Supplementary Figures (Figs S1-10)

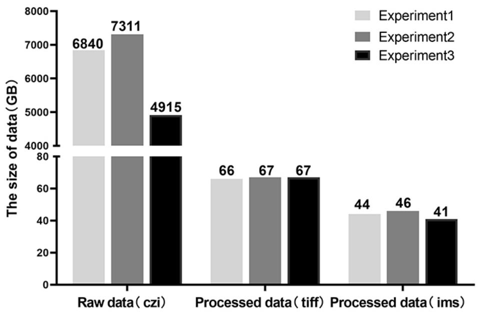

Supplementary Fig. S1 The Size of light-sheet imaging data decreases ~100 fold after AFEIO processing. The datasets collected from three independent imaging experiments, showing that the raw data in Carl Zeiss image (czi) format is ~5-7 TB, and the data processed by AFEIO in tiff format is <70 GB or in Imaris (ims) format is <50 GB.

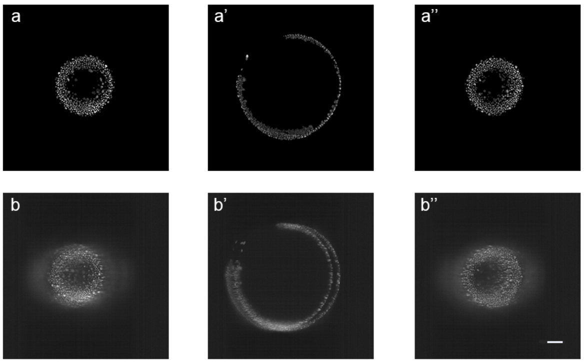

Supplementary Fig. S2 The image fusion by AFEIO (two-view imaging) results in higher resolution images than by Zen (four-view imaging). Three different planes were either fused by AFEIO (a, a’, aa’’) or by Zen (Zeiss) (b, b’, b’’); note that AFEIO, but not Zen, generated high-quality image fusion.

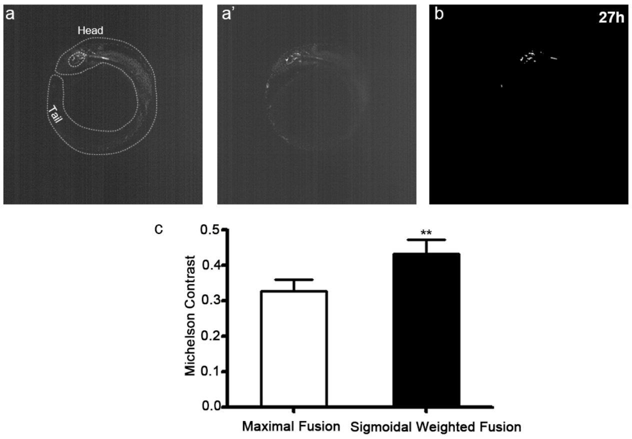

Supplementary Fig. S3 Image processing by AFEIO. (a, a’) Original Tg(kdrl:mCherry) images of embryo at 27 hpf, illuminated from the right (a) and left (a’). (b) The processed image after fusion and thresholding. (c) Michelson contrast with maximal fusion and sigmoidal weighted fusion; note that the latter significantly improves the contrast ($p = 0.0094$; $n = 5$).

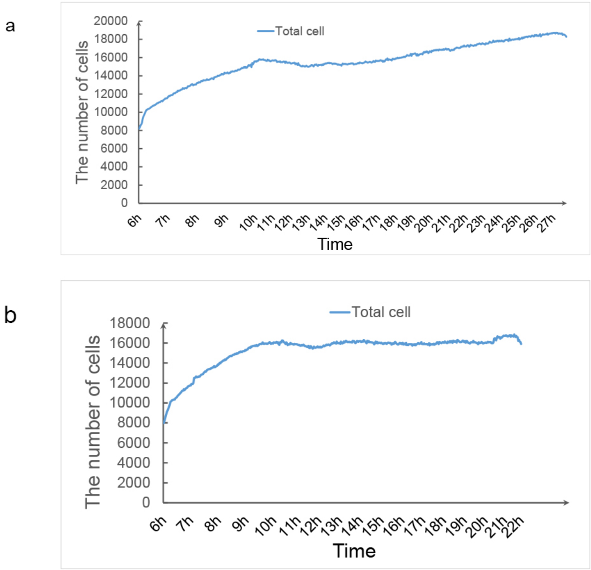

Supplementary Fig.S4 Dynamic changes in the total number of embryonic cells during early embryogenesis. The total numbers of cells were ~8000 at 6 hpf, increased quickly until 10 hpf, then decreased from 10 to 12 hpf, probably due to apoptosis, and later increased slowly until the end of imaging in embryo #1 (a) and embryo #2 (b).

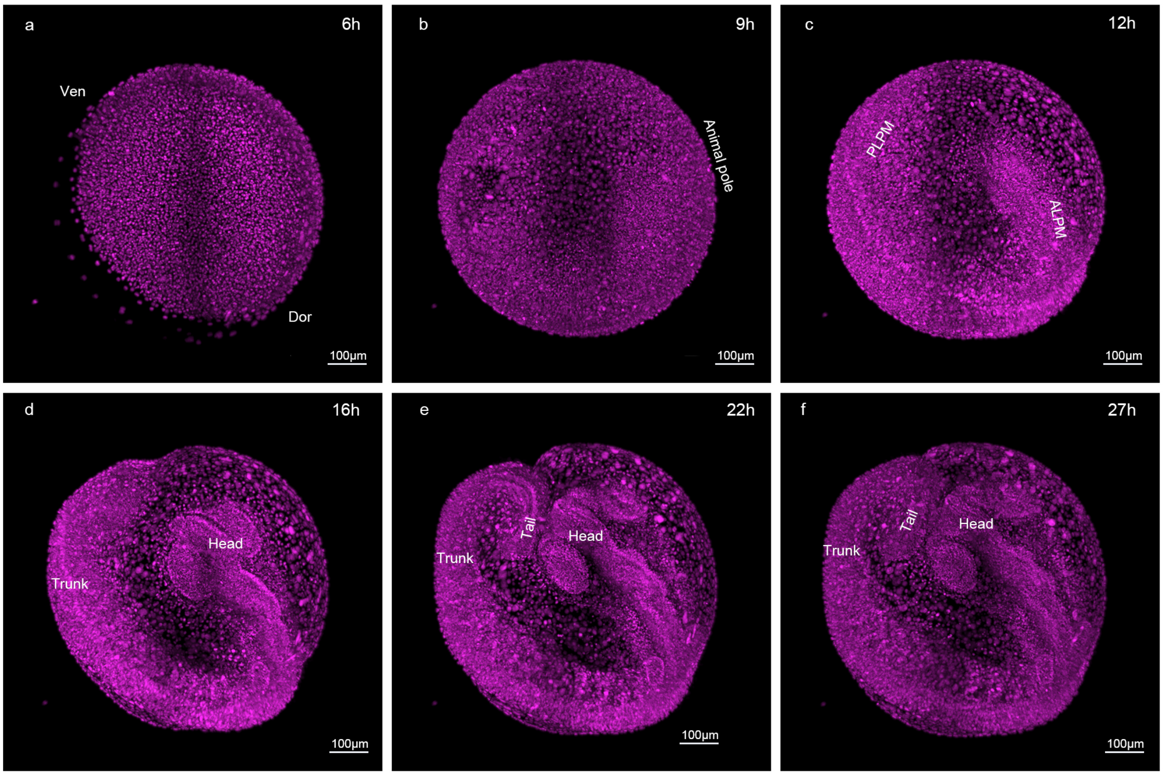

Supplementary Fig. S5 Developmental morphogenesis of embryo #1 from 6 to 27 hpf (also shown in Supplementary Movie S1). (a–f) Side view of the embryo at 6 hpf (a), 9 hpf (b), 12 hpf (c), 16 hpf (d), 22 hpf (e) and 27 hpf (f); note that the embryo develops normally but is not able to stretch out, with the growing tail bent (e–f) because it is embedded in FEP at an inner diameter of only 0.8 mm. Dor, dorsal; Ven, ventral; ALPM, anterior lateral plate mesoderm; PLPM, posterior lateral plate mesoderm. Scale bars, 100 μm.

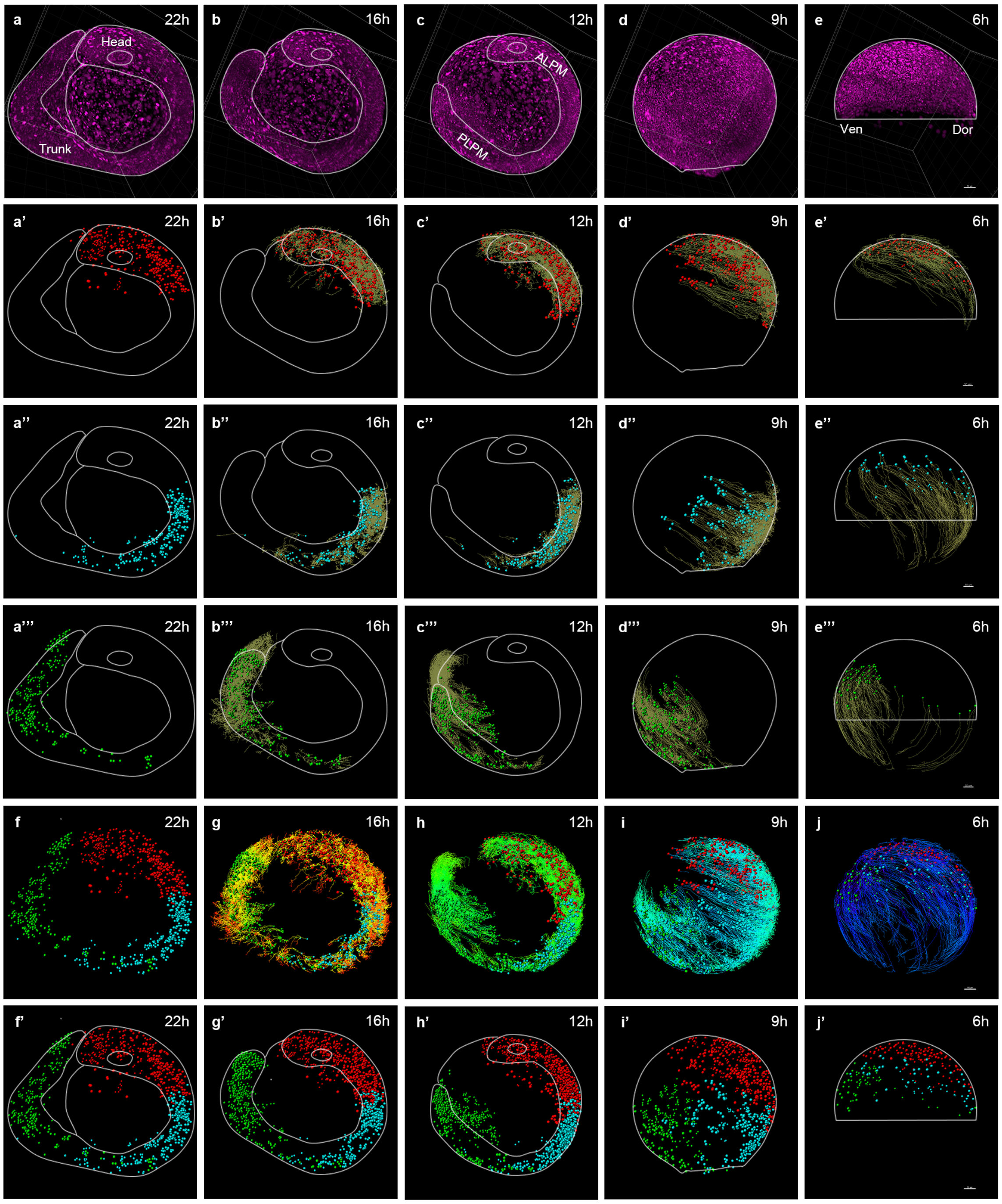

Supplementary Fig. S6 Retrospective cell-lineage tracing creates a comprehensive map of the origin of vascular endothelial cells along the dorsal–ventral and anterior–posterior axes of the gastrula in embryo #2. (a–e) Developmental morphogenesis of embryo #2 at 22 hpf (a),16 hpf (b), 12 hpf (c), 9 hpf (d), and 6 hpf (e). (a'–e') Retrospective lineage tracing of head endothelial cells at 22 hpf (a'), 16 hpf (b'), 12 hpf (c'), and 9 hpf (d') to the gastrula

progenitors at 6 hpf (e’). (a”–e”) Retrospective lineage tracing of anterior trunk endothelial cells at 22 hpf (a”), 16 hpf (b”), 12 hpf (c”), and 9 hpf (d”) to gastrula progenitors at 6 hpf (e”). (a’”–e’”) Retrospective lineage tracing of posterior trunk endothelial cells at 22 hpf (a’”), 16 hpf (b’”), 12 hpf (c’”), and 9 hpf (d’”) to the gastrula progenitors at 6 hpf (e’”). (f–j) Retrospective lineage tracing showing the distribution of the three clusters of endothelial cells (red, blue, and green) with lineage tracking lines (multiple-colored lines) from 22 hpf (f), 16 hpf (g), 12 hpf (h), and 9 hpf (i) to gastrula progenitors at 6 hpf (j). (f’–j’) Retrospective lineage tracing showing the distribution of the three clusters of endothelial cells (red, blue, and green) without lineage tracking lines from 22 hpf (f’), 16 hpf (g’), 12 hpf (h’), and 9 hpf (i’) to the gastrula progenitors at 6 hpf (j’). Scale bars, 50 μm.

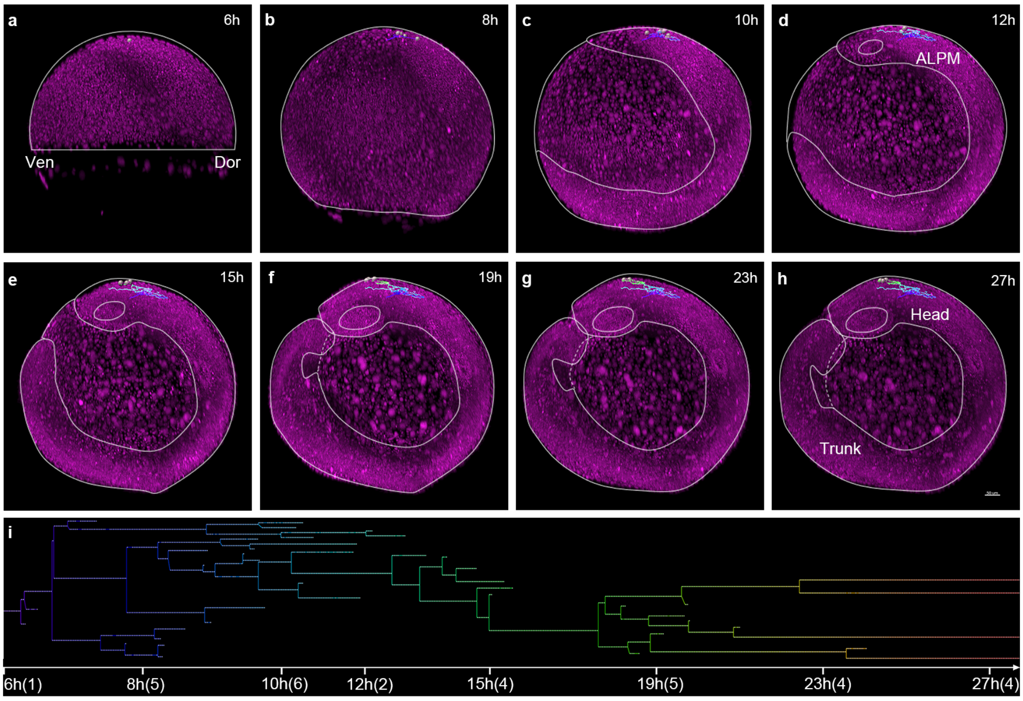

Supplementary Fig. S7 Cell-lineage tracing of early zebrafish embryos at single-cell resolution. (a, b) A selected cell near the animal pole at 6 hpf (a) divides and migrates upwards to the animal pole, and then migrates to the right side of the embryo at ~8 hpf (b). (c, d) These descendants begin to migrate back at 9 hpf, a group of descendants disappears, and the remaining group continues to migrate to the upper left of the starting position at 12 hpf. (e–h) These descendants migrate further and finally reach the top of the head between the eyes (dynamic lineage tracking in Supplementary Movie S16). (i) The cell-lineage tree map from a single gastrula cell in the animal pole at 6 hpf to four head endothelial cells at 27 hpf; note that some descendants disappear during development. Scale bar, 50 μm.

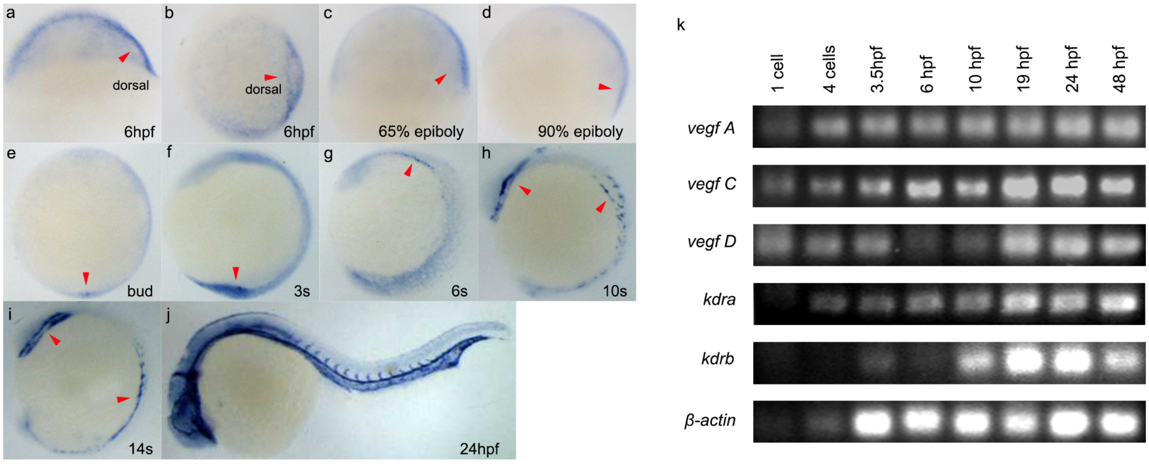

Supplementary Fig. S8 *kdrl* is enriched in the dorsal gastrula and blood vessels during early somitogenesis in zebrafish. (a–j) *In situ* RNA hybridization with the *kdrl* probe in embryos at 6 hpf (a, b), 65% epiboly (c), 90% epiboly (d), bud stage (e), 3 somites (f), 6 somites (g), 10 somites (h), 14 somites (i), and 24 hpf (j). Note the highest *kdrl* expression in the dorsal region (a, b, arrowheads), and in the ALPM and PLPM regions (g–i, arrowheads), and blood vessels (j). In a and c–j, lateral views are shown with anterior to the left. In b, top views are shown with dorsal to the right. Scale bar, 100 μm. (k) Expression of *vegfA*, *vegfC*, *vegfD*, *kdrl*, and *kdr* in 1-cell, 4-cell, 3.5-hpf, 6-hpf, 10-hpf, 19-hpf, 24-hpf, and 48-hpf embryos by semi-quantitative RT-PCR. Note that all three VEGF ligand genes (vegfA, C, and D) and the VEGF receptor kdrl had maternal expression starting from 1-cell to 48-hpf embryos, and the VEGF receptor kdr had no maternal expression starting from 3.5 to 48 hpf. β-actin was used as an internal control.

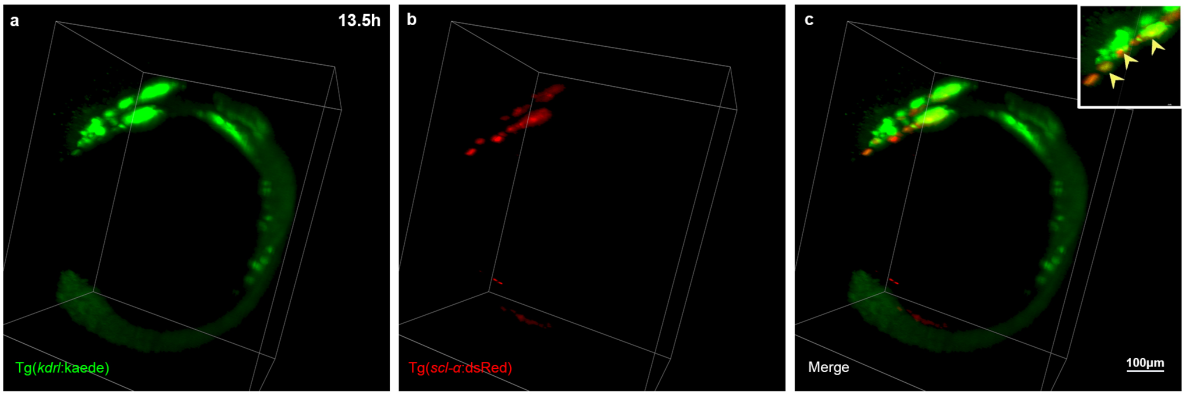

Supplementary Fig. S9 The expression of Tg(*kdrl*:kaede) is partly co-localized with Tg(*scl-α*:dsRed) in the anterior lateral plate mesoderm. (a) Image on the Tg(*kdrl*:kaede) green channel at 13.5 hpf. (b) Image on the Tg(*scl-α*:dsRed) red channel at 13.5 hpf, noting that the anterior lateral plate mesoderm was clearly labelled. (c) The merged image at 13.5 hpf. Scale bar, 50 μm.

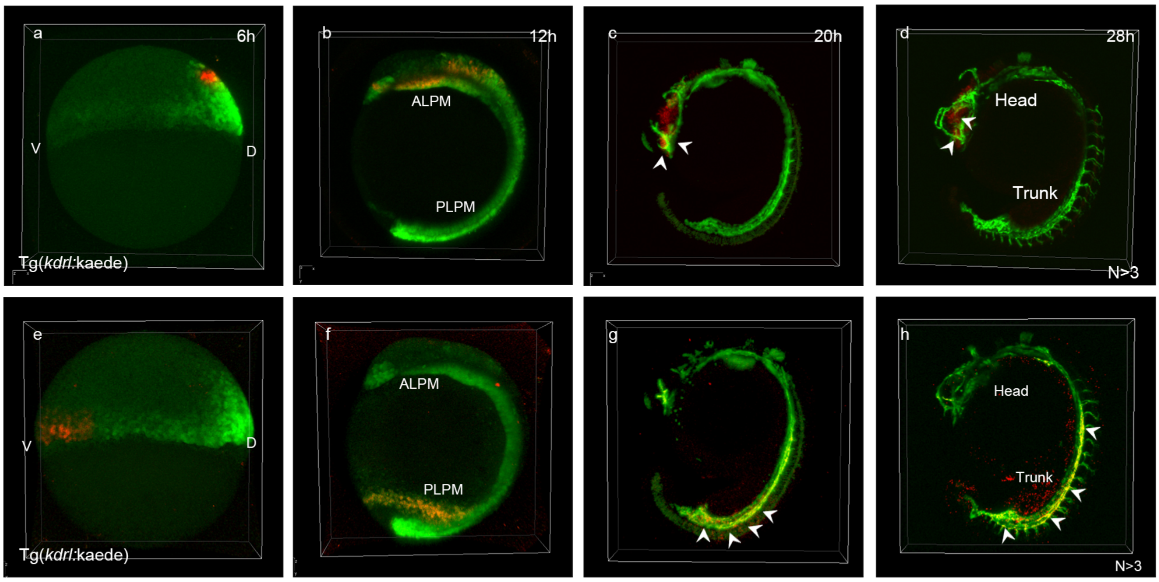

Supplementary Fig. S10 Prospective lineage tracing delineates the distinct origins of vascular ECs in the head and trunk regions. (a) Several Kaede+ cells in the dorsal region were converted to red by 405-nm laser excitation. (b–d) Red cells reached the anterior lateral plate mesoderm (ALPM) at 12 hpf (b) and migrated to the eyes (arrowheads) at 20–28 hpf (c, d). (e) Several Kaede+ cells in the ventral-lateral region were converted to red by 405-nm laser excitation. (f–h) Red cells approached the posterior lateral plate mesoderm (PLPM) at 12 hpf (f) and migrated to form trunk ECs at 20 hpf (g) and 28 hpf (h). Arrowheads point to yellow ECs; n >3.

Supplementary Movies

The movies can be downloaded from the below website:

https://disk.pku.edu.cn:443/link/4ED92786A171450C943A370BBC545007

or viewed from the below website:

https://www.youtube.com/playlist?list=PLK9xesWj3KRk3rt0nshxQOCokbqCSI2_P

Movie S1

Developmental morphogenesis of embryo #1 with a vegetal pole view from 6 to 27 hpf (magenta, the EGFP channel for labeling all cell nuclei); note that the embryo develops normally but is not able to stretch out, and the growing tail bends after ~19 hpf because it is embedded with FEP at an inner diameter of only 0.8 mm.

Movie S2

Developmental morphogenesis of embryo #2 with the animal pole on the top from 6 to 22 hpf (magenta, EGFP channel for labeling all cell nuclei); note that the embryo develops normally but is not able to stretch out, and the growing tail bends after ~19 hpf, because it is embedded with FEP at an inner diameter of only 0.8 mm.

Movie S3

Developmental morphogenesis of embryo #3 with the animal pole at the bottom from 7 to 27 hpf (magenta, EGFP channel labeling all cell nuclei); note that the embryo develops normally but is not able to stretch out, and the tail grows under the head after ~19 hpf, because it is embedded with FEP at an inner diameter of only 0.8 mm.

Movie S4

Representative movie showing the nuclei of embryo #1 at 6.25 hpf (magenta, nuclear EGFP signals of all cells; green, nuclear spots identified by Imaris). As the embryo rotates, the video shows an enlarged view of the nuclear EGFP signals at different locations.

Movie S5

Representative movie showing the nuclei of embryo #1 at 16.7 hpf (magenta, nuclear EGFP signals of all cells; green, nuclear spots identified by Imaris). As the embryo rotates, the video shows an enlarged view of the nuclear EGFP signals at different locations.

Movie S6

An embryo imaged from 6 to 20 hpf using the Luxendo Muvi-SPIM and the image dataset processed by AFEIO software (magenta, nuclear EGFP signals of all cells).

Movie S7

Representative movie showing the maximum-intensity projections of a light-sheet time-lapse recording of embryo #1 from 6 to 27 hpf (colored lines, global cell tracks including cell movements and cell divisions; darker colors, tracks of cell positions and movements in the embryo at older time points; brighter colors, tracks and movements at younger time points). The track duration displayed is from 6 hpf to the time of recording.

Movie S8

Retrospective lineage tracing of vascular endothelial cells in the head from 27 hpf to gastrula progenitors at 6 hpf. Representative optical images from this movie are shown in Fig. 3a–h. Magenta, nuclei; green, selected cells; colored lines, cell tracks including cell divisions and movements. The track duration displayed is from 27 hpf to the time of recording.

Movie S9

Retrospective lineage tracing of vascular endothelial cells in the posterior trunk from 27 hpf to gastrula progenitors at 6 hpf. Representative optical images from this movie are shown in Fig. 3j–q. Magenta, nuclei; green, selected cells; colored lines, cell tracks including cell divisions and movements. The track duration displayed is from 27 hpf to the time of recording.

Movie S10

Retrospective fate mapping of the head vascular endothelial cells of embryo #1 from 27 hpf to dorsal-anterior gastrula progenitors at 6 hpf (red, vascular endothelial cells of the head; colored lines, retrospective tracks of vascular endothelial cells). The displayed track duration contains 100 time points.

Movie S11

Retrospective fate mapping of anterior trunk endothelial cells of embryo #1 from 27 hpf to dorsal-lateral gastrula progenitors at 6 hpf (blue, anterior trunk endothelial cells; colored lines, retrospective tracks of vascular endothelial cells). The displayed track duration contains 100 time points.

Movie S12

Retrospective fate mapping of posterior trunk endothelial cells of embryo #1 from 27 hpf to posterior-lateral gastrula progenitors at 6 hpf (green, posterior trunk endothelial cells; colored lines, retrospective tracks of vascular endothelial cells). The displayed track duration contains 100 time points.

Movie S13

In toto imaging of the migration and division of gastrula endothelial precursors at 6 hpf to all vascular endothelial cells at 27 hpf (red, vascular endothelial cells of the head; blue, endothelial cells of the anterior trunk; green, endothelial cells of the posterior trunk; colored lines, cell movements and divisions of endothelial precursors; darker colors, cell positions and movements at older time points; brighter colors, cell positions and movements at younger time points. The track duration displayed is from 6 hpf to the time of recording.

Movie S14

Retrospective fate mapping of vascular endothelial cells of embryo #1 from 27 hpf to gastrula progenitors at 6 hpf (red, vascular endothelial cells of the head; blue, endothelial cells of the anterior trunk; green, endothelial cells of the posterior trunk. The track duration contains 100 time points.

Movie S15

Retrospective fate mapping of vascular endothelial cells of embryo #1 from 27 hpf to gastrula progenitors at 6 hpf (red, vascular endothelial cells of the head; blue, endothelial cells of the

anterior trunk; green, endothelial cells of the posterior trunk. The track displayed is from 27 hpf to the time of recording.

Movie S16

Developmental tracks of a single progenitor cell in the animal pole from 6 hpf to head endothelial cells at 27 hpf. Representative optical images of this movie are shown in Supplementary Fig. S7. Magenta, nuclear signals; green, selected cells; colored lines, tracks including cell movement and cell divisions; darker colors, cell positions and movements at older time points; brighter colors, cell positions and movements at younger time points. The track duration displayed is from 6 hpf to the time of recording.
